# Postural Syncope and Constipation: An Unusual Presentation of a Duodenal Dieulafoy's Lesion

**DOI:** 10.1155/2017/6983434

**Published:** 2017-03-07

**Authors:** Ahmed Dirweesh, Alvarez Chikezie, Muhammad Yasir Khan, Sana Zia, Muhammad Tahir

**Affiliations:** Department of Internal Medicine, Seton Hall University School of Health and Medical Sciences, Saint Francis Medical Center, Trenton, NJ, USA

## Abstract

Dieulafoy lesions are a rare etiology of gastrointestinal bleeding from a large caliber-persistent tortuous submucosal artery. They account for 1-2% of all causes of acute gastrointestinal hemorrhage with 80%–95% of these lesions located in the stomach along the lesser curvature. One-third of these lesions present at an extragastric location, with the proximal duodenum accounting for 15% of them. We present a 21-year-old male with no significant past medical history or risk factors, who presented with repeated syncopal episodes followed by hematemesis, found to have a Dieulafoy lesion located at the duodenal bulb. This lesion was diagnosed and successfully treated via upper endoscopy with epinephrine injection and the application of 2 endoscopic clips.

## 1. Introduction

Dieulafoy lesion represents an uncommon source of gastrointestinal (GI) bleeding but a potentially fatal one. It is defined anatomically as a dilated, aberrant, submucosal artery that erodes overlying gastrointestinal mucosa in the absence of an underlying ulcer or aneurysm [1]. Approximately 70% of these lesions occur within 6 cm from the gastroesophageal junction and along the lesser gastric curve but can be found anywhere along the GI tract [1]. We report a case of a 21-year-old male with no significant past medical history or risk factors, who presented with repeated syncopal episodes followed by acute upper gastrointestinal bleeding due to an actively bleeding duodenal Dieulafoy lesion which was diagnosed and successfully treated via upper endoscopy.

## 2. Case Presentation

**Figure 1:**
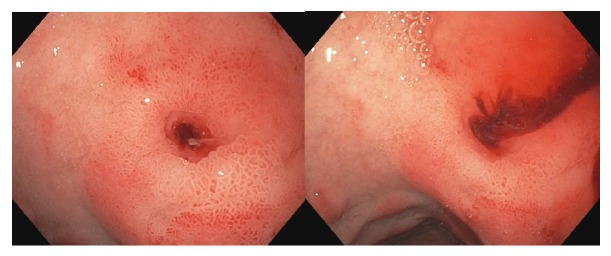
Dieulafoy lesion with active bleeding.

A 21-year-old male with no significant past medical history presented to the emergency department after having multiple episodes of syncope upon standing associated with prodromal symptoms but with no loss of bowel function, urinary incontinence, or palpitations. He did not experience any seizures or history of seizures. Patient also reported having 3 weeks of worsening lower vague abdominal pain and constipation with flatus prior to presentation. He denied any prior nausea or emesis but did develop an episode of nausea with coffee-ground emesis in the emergency department. There was no history of alcohol abuse or NSAID use. On admission, his orthostatics were positive and respiratory rate was 17 breaths per minute with a heart rate of 113 beats per minute. There were no significant findings of physical exam apart from conjunctival pallor, tachycardia, and lower abdominal tenderness without guarding, rigidity, or rebound. Hemoglobin and hematocrit were initially 11 g/dL and 34.2%, respectively, with a significant subsequent decline to 6.0 g/dL and 18.2%, respectively, with positive occult stool suggestive of gastrointestinal hemorrhage. Two units of packed red blood cells were given along with 2 L normal saline bolus IV. Coagulation parameters were within normal limits. Patient was transferred to the ICU and subsequently underwent esophagogastroduodenoscopy, which revealed an actively bleeding Dieulafoy lesion located in the duodenal bulb (Figure 1).

**Figure 2:**
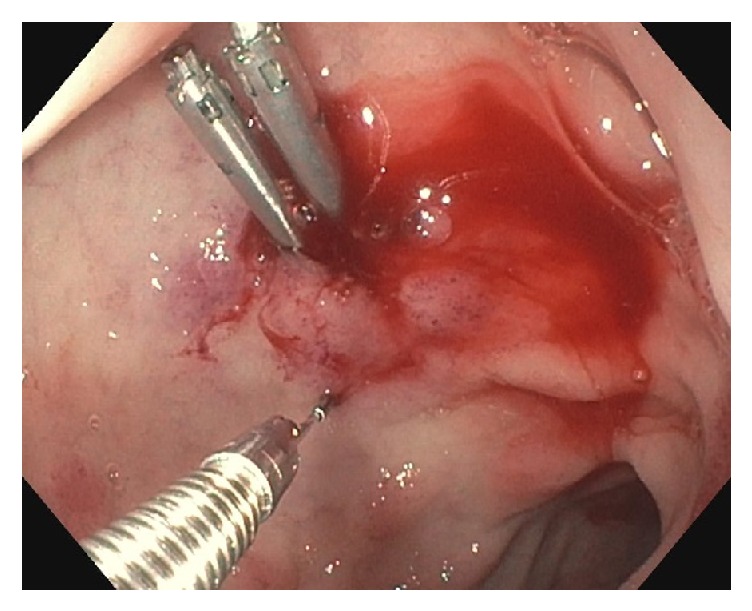
Two endoclips applied to the bleeding vessel.

Homeostasis was successfully achieved with epinephrine injection and the application of 2 endoscopic clips (Figure 2).

A repeat esophagogastroduodenoscopy revealed normal esophageal and gastric mucosa and a Dieulafoy lesion in the duodenal bulb with 2 endoclips in place. The patient tested negative for* H. pylori* and the rest of his hospital course was uneventful.

## 3. Discussion

Dieulafoy lesion is a rare but potentially fatal etiology of gastrointestinal bleeding due to its ability to cause significant acute life threatening hemorrhage. It consists of a large caliber-persistent tortuous submucosal artery [2]. As the artery traverses its course, it fails to taper as it approaches the mucosa and hence has a diameter of 1 to 5 mm [2]. The artery extends through a small mucosal defect that is typically 2 to 5 mm long and does not show any other abnormality of the arterial wall along with a histologically normal surrounding mucosa [2]. This caliber artery is up to ten times larger than the maximal caliber of normal submucosal vessels. Due to the abnormal exposure of this vessel, even minor mechanical trauma from a food bolus can lead to erosion of this artery into the lumen causing severe acute GI hemorrhage. Dieulafoy lesions are the source of 1-2% of acute gastrointestinal hemorrhage, with 80%–95% of these lesions located in the stomach within 6 cm of the gastroesophageal junction along the lesser curvature [3]. Approximately one-third of these lesions present at an extragastric location, with the proximal duodenum accounting for 15% of them, followed by the colon at 2% [3]. Dieulafoy lesions can occur at any age with a mean onset in the fifth decade, twice as common in men as women with no specific familial predisposition, and are more common in patients with NSAID, aspirin, or antiplatelet use or multiple comorbidities such as cardiovascular disease, renal disease, hypertension, and diabetes mellitus [2]. A recent case control study, which looked at the risk factors associated with Dieulafoy lesion formation in the upper gastrointestinal tract in 42 subjects, found that antiplatelet agents and alcohol consumption were risk factors associated with Dieulafoy lesion formation [4]. Our patient was atypical for this presentation due to his young age and absence of any comorbidities, NSAID, alcohol abuse, or antiplatelet use. Patients are usually asymptomatic without preceding symptoms before presenting with acute GI hemorrhage, which manifests as hematochezia, melena, or hematemesis. In one study, which reviewed 177 cases, 51% presented with hematemesis and melena, 28% of patients presented with hematemesis, and 18% presented with melena alone [5].

Histologically, there is subintimal fibrosis of the artery without any structural aneurysmal change and without any inflammation at the edge of the mucosal defect, which is in stark contrast to that which is present in peptic ulcer disease [6]. The diagnostic modality of choice depends on the clinical presentation with upper GI endoscopy and push enteroscopy being the most commonly used means of detection and treatment. Reported success rates of endoscopy are in excess of 90% [3]. Angiography is useful when endoscopy fails to localize the lesion [2]. Advancements in endoscopic techniques have significantly reduced the need for surgical intervention. The endoscopic criteria needed for diagnosis include (1) active arterial spurting or micropulsatile streaming from a minute (<3 mm) mucosal defect or through normal surrounding mucosa; (2) visualization of a protruding vessel with or without active bleeding within a minute mucosal defect or through normal surrounding mucosa; and (3) the appearance of a fresh, densely adherent clot with a narrow point of attachment to a minute mucosal defect or mucosa of normal appearance [1]. There are three major methods of achieving homeostasis via endoscopic techniques. They include the following: (1) regional injection via local epinephrine injection and sclerotherapy; (2) thermal: via electrocoagulation, heat probe coagulation, and argon plasma coagulation (APC); and (3) mechanical: banding and hemostatic clips [2].

There is currently no consensus on the treatment of Dieulafoy lesions. The literature suggests that combination endoscopic therapy is superior to monotherapy consisting of injection therapy followed by thermal or mechanical therapy, with permanent hemostasis achieved in 95% of all cases [2]. The risk of rebleeding from Dieulafoy's lesions is reported to be 9% to 40% with the recurrence rate of bleeding being higher in endoscopic monotherapy compared with combination therapy [2]. In one clinical trial, which looked at endoscopic therapy in 24 patients with Dieulafoy's lesions, initial hemostasis was achieved in 91.7% of patients undergoing mechanical therapy and 75% of those undergoing injection therapy with the rate of recurrent bleeding in the mechanical therapy group being significantly lower in comparison to the injection therapy group (8.3% versus 33.3%, *p* < 0.05) [7]. The results of one retrospective study showed that endoscopic thermal coagulation with or without epinephrine injection should be the initial treatment of choice for Dieulafoy's lesions [8]. In one small prospective, randomized trial of 26 patients, there were no detectable differences in the efficacy or the safety of endoscopic band ligation and endoscopic hemoclip placement in the management of bleeding gastric Dieulafoy's lesions [9]. In the end, the treatment modality chosen should be tailored to each individual case with emphasis placed on the mode of presentation, the site of the lesion, and the available expertise. In our case, homeostasis was successfully achieved with epinephrine injection and the application of 2 endoscopic clips without evidence of subsequent rebleeding on repeat endoscopy.

In conclusion, one must always be mindful to keep Dieulafoy lesion as part of their differential diagnosis when evaluating any individual of any age who presents with acute gastrointestinal hemorrhage, even in the absence of significant comorbidities or risk factors. Early diagnosis is crucial, with endoscopy being the investigative modality of choice. The specific treatment of choice should be individualized to each patients' clinical presentation, site of lesion, and the technical expertise of the operator.
